# Dynamic but constrained: repeated acquisitions of nutritional symbionts in bed bugs (Heteroptera: Cimicidae) from a narrow taxonomic pool

**DOI:** 10.1128/msystems.01247-25

**Published:** 2025-11-10

**Authors:** Václav Hypša, Jana Martinů, Sazzad Mahmood, Shruti Gupta, Eva Nováková, Steffen Roth, Ondřej Balvín

**Affiliations:** 1 Department of Parasitology, Faculty of Science, University of South Bohemia 48271, České Budějovice, Czech Republic; 2 Institute of Parasitology, Biology Centre, ASCR, v.v.i., České Budějovice, Czech Republic; 3 University Museum of Bergen 87341 , Bergen, Norway; 4 Department of Ecology, Faculty of Environmental Sciences, Czech University of Life Sciences Prague 48371, Prague, Czech Republic; The Pennsylvania State University, State College, Pennsylvania, USA

**Keywords:** symbiosis, bed bugs, coevolution, genomics, microbiome

## Abstract

Bed bugs (Heteroptera: Cimicidae) harbor obligate bacterial symbionts that supplement their blood diet with missing nutrients, especially B vitamins. The primary symbiont, transovarially transmitted *Wolbachia*, is notable for a horizontally acquired biotin operon. Additional maternally inherited bacteria, including *Symbiopectobacterium* and *Tisiphia*, have been detected but are considered facultative and nonessential. However, nearly all current knowledge is derived from the human-associated *Cimex lectularius*, leaving symbiont diversity across more than 100 bed bug species largely unknown. Using amplicon and metagenomic data, we identified *Wolbachia*, *Symbiopectobacterium*, *Sodalis*, *Serratia*, and *Tisiphia* as candidate symbionts, with at least 16 independent acquisition events across the cimicid species, sometimes involving multiple strains per host. Phylogenetic comparisons indicated that some of these origins were followed by cospeciation. *Wolbachia* was present in most hosts except Cacodminae, where *Symbiopectobacterium* occurred as the sole symbiont, suggesting its obligate role. Analysis of 23 draft genomes revealed heterogeneity in size and gene content, consistent with varying stages of symbiotic reduction. Most lineages lost many biosynthetic pathways; only riboflavin and lipoic acid synthesis remained universally conserved. Our survey reveals a dynamic evolution of bed bug symbioses, with repeated symbiont acquisitions, cospeciation, and frequent coinfections. Despite independent origins, most symbionts belong to *Wolbachia*, *Symbiopectobacterium*, or *Sodalis*, implying unknown mechanisms shaping host specificity. Two points merit further study. First, *Symbiopectobacterium* as the sole obligate symbiont in Cacodminae suggests broader sampling may uncover greater symbiotic diversity. Second, uncertainties in biotin synthesis function call for deeper investigation into the evolution of this pathway in symbiotic bacteria.

**IMPORTANCE:**

Bed bugs are obligate blood-feeding insects that depend on bacterial partners to supply nutrients missing from their diet. Most previous research has focused on the human-associated species *Cimex lectularius*, leaving little known about symbiont diversity across other species. By surveying a broad phylogenetic range, we found that bed bugs have repeatedly acquired different bacteria as symbionts, including lineages not previously recognized as essential. Notably, finding *Symbiopectobacterium* as the sole symbiont in one subfamily shows that the nutritional partnerships in bed bugs are more dynamic than previously thought. At the same time, the majority of the 16 independent acquisitions involve only four bacterial genera, suggesting efficient mechanisms that constrain and shape bed bug-symbiont specificity.

## INTRODUCTION

Many insect groups maintain obligate associations with symbiotic bacteria that supplement essential nutrients missing from their diets (1). While some of these associations exhibit remarkable long-term stability (e.g., the *aphid–Buchnera* relationship, which has persisted over a hundred million years of cospeciation; ref 2), others undergo a dynamic process of symbiont acquisition, loss, and replacement (3). The duration and intimacy of these associations are typically reflected in characteristic changes to symbiont genomes, including genome size reduction, loss of metabolic functions, and shift in nucleotide composition toward AT (4). In insects that feed exclusively on vertebrate blood, the presence of obligate symbionts is critical for host viability and fitness as these symbionts provide B vitamins that are deficient in the blood diet (5). Studies across various hematophagous insects have shown that these obligate mutualists originate from a diverse array of bacterial taxa (6).

One such exclusively hematophagous group is bed bugs, the heteropteran family Cimicidae, which comprises over one hundred described species that are associated primarily with bats and birds (7). However, for practical reasons, the most extensively studied cimicid species is the human-associated common bed bug (*Cimex lectularius*). Three bacterial groups have been demonstrated or suggested to be transovarially transmitted symbionts in this species. The most prominent strain is the widespread insect symbiont *Wolbachia*. This bacterium, best known for manipulating insect reproduction (8), was first identified in two cimicid species by Rasgon and Scott (9), and its role as an obligate symbiont in *C. lectularius* was subsequently documented by several studies (10–13). The other two symbionts are considered facultative and likely nonessential. The first is a bacterium related to the symbiont of *Euscelidius variegatus*, which was initially described from *Cimex lectularius* (14) and was later shown to belong to the genus *Symbiopectobacterium* (15). The second is *Rickettsia*-related *Candidatus* Tisiphia (16, 17). The significance of these two presumably facultative symbionts for host fitness remains unclear.

Several lines of evidence strongly support the view that *Wolbachia* are obligate symbionts essential for bed bug development and reproduction: their ubiquity across bed bug populations, efficient transovarial transmission (10, 17), and the presence of a horizontally acquired biotin operon (11). Furthermore, direct experimental evidence of the nutritional role of *Wolbachia* in *C. lectularius* has been provided by multiple studies (10, 11, 18). A typical consequence of such an intimate host‒symbiont association is a shared coevolutionary history, reflected in congruent host and symbiont phylogenies. A screening study by Balvin et al. (19) indeed revealed a partial coevolutionary signal between *Wolbachia* and their Cimicinae hosts. However, this cospeciation pattern did not include all Cimicidae-associated *Wolbachia*, suggesting that symbiont evolution within this host group is more complex than a model of strict vertical transmission and coevolution. This finding aligns with earlier results of Sakamoto, Feinstein, and Rasgon (20), who detected *Wolbachia* strains from two different supergroups, F and A, in cimicid museum samples. Furthermore, in *C. lectularius*, genome-wide analysis has revealed extensive lateral gene transfer from several bacterial genera, including the known insect symbionts *Arsenophonus*, *Sodalis*, and *Hamiltonella* (21). These genetic remnants may represent relics of ancient, now-lost symbiotic associations, as suggested in other insect systems (22). Indirect support for this hypothesis of dynamic evolution in cimicid‒symbiont associations comes from comparisons with other obligate hematophages, which harbor diverse microbiomes with complex evolutionary histories (23, 24).

To test the hypothesis that symbioses in Cimicidae have followed a complex and dynamic evolutionary trajectory characterized by multiple independent origins across diverse bacterial genera, we analyzed a broad set of cimicid specimens via both amplicon and metagenomic sequencing. Amplicon sequencing serves primarily as a screening tool to assess the distribution of symbiotic taxa across the Cimicidae phylogeny. Metagenomic sequencing was used to explore the genomic characteristics and metabolic capacities of the symbionts, with a focus on 14 samples representing 13 species from five subfamilies: Afrocimicinae, Haematosiphoninae, Cacodminae, Cimicinae, and the basal Primicimicinae.

## RESULTS AND DISCUSSION

### Amplicon screening

**Fig 1:**
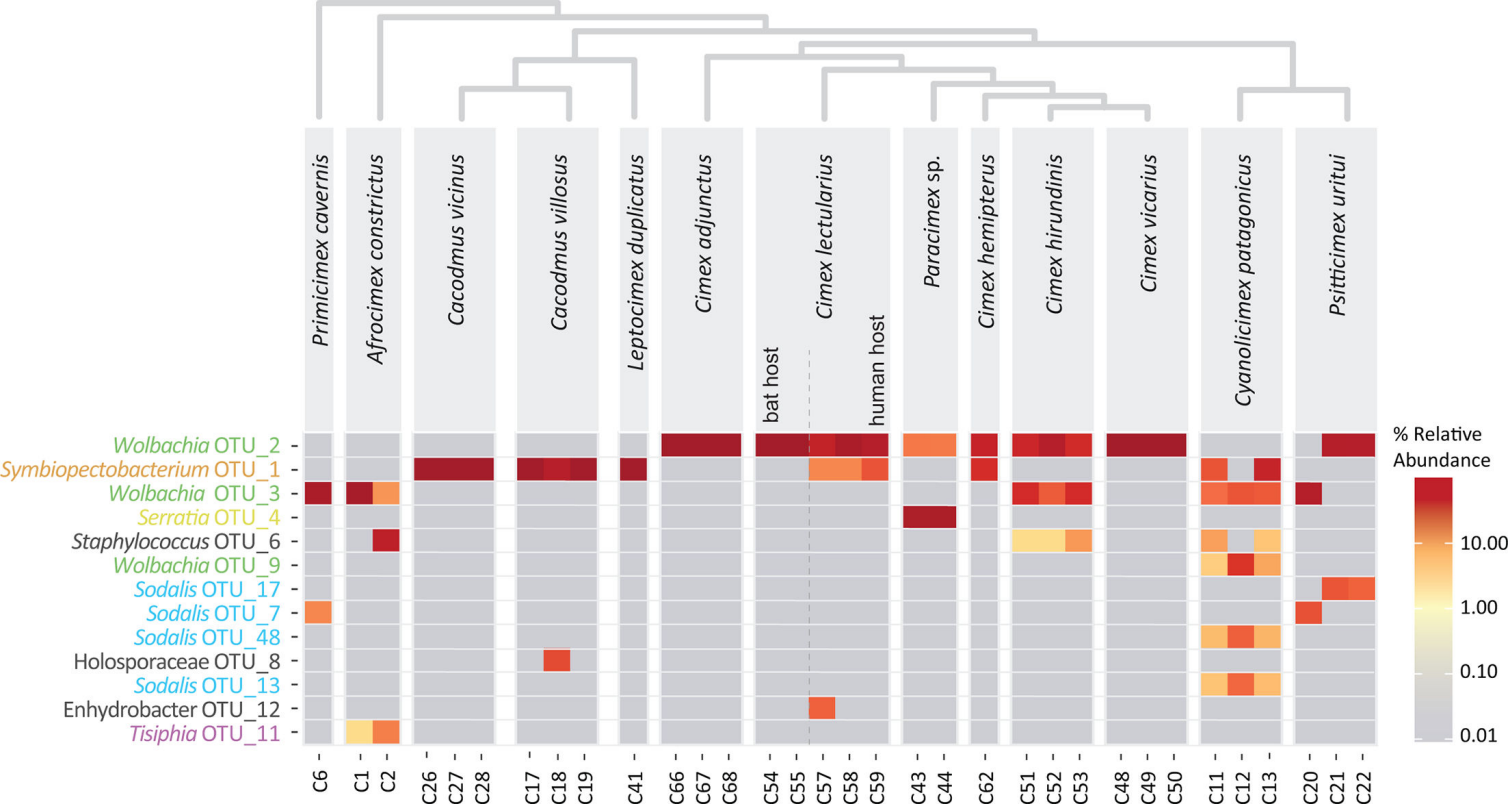
Distribution of 13 most abundant OTUs across 33 cimicid samples. Symbiotic taxa are color-coded to highlight their (co-)occurrence and relative abundance within the data set. Phylogenetic relationships among the species are shown on the top.

An initial overview of the symbiotic interactions in 13 Cimicidae species was obtained through 16S rRNA gene barcoding. The dominant operational taxonomic units (OTUs) correspond to symbiotic taxa and, to some extent, allow differentiation among phylogenetically distant lineages within a single genus (Fig. 1). The most abundant taxon, found in 10 out of 13 species, was assigned to the genus *Wolbachia,* represented by three OTUs in our data set. The nucleotide identity of the best BLAST hits suggested that these OTUs likely corresponded to different *Wolbachia* symbionts from three supergroups (*Wolbachia* F, E, and B; Table S1). The distribution pattern of these OTUs (Fig. 1) further suggested dual *Wolbachia* infection for at least two host species, *Cimex hirundinis* and *Cyanolicimex patagonicus*. The genus *Symbiopectobacterium* is represented by a single OTU dominating the microbiomes of *Cacodmus* spp. and *Leptocimex duplicatus*. This *Symbiopectobacterium* OTU is also detected alongside *Wolbachia* in *Cimex hemipterus* and *Cimex lectularius* samples collected from human hosts. In contrast, the distributions of *Serratia* and *Tisiphia* (torix Rickettsia) symbionts are rather host-specific, with each being restricted to a single species in our data set, i.e., *Paracimex* sp. and *Afrocimex constrictus*, respectively. The last symbiotic taxon identified, belonging to the genus *Sodalis*, is represented by four OTUs and has the most complex distribution pattern. Two *Sodalis* OTUs associated with *Cyanolicimex patagonicus* displayed surprisingly low nucleotide identity (92.75%). In *the P. uritui* microbiome, the other two *Sodalis* OTUs varied among the screened individuals. The presence of multiple OTUs for the same symbiotic taxon can, in principle, reflect either a true biological pattern or a methodological artifact (e.g., sequencing errors). However, because our amplicon data were processed via a highly conservative pipeline that retains only the highest-quality reads (see Materials and Methods), a biological explanation is more likely. The observed diversity may thus result from coinfection by distinct symbiont lineages or from intragenomic heterogeneity of 16S rRNA gene copies, especially in *Sodalis* symbionts known to carry up to seven rRNA operons (25). Since nucleotide identities among OTUs for both *Sodalis* and *Wolbachia* symbionts range from 92.75% to 96.75%, we propose that these OTUs represent distinct symbiotic lineages.

### Metagenomic assemblies and drafts of symbiont genomes

The Illumina short-read sequencing data obtained for 14 Cimicidae samples (13 species), along with the main characteristics of their metagenomic assemblies, are summarized in Table S2. The screening of these assemblies via bacterial gene queries led to the identification of 23 genome drafts corresponding to known symbiotic taxa, as indicated by the results of the amplicon screening (Table 1). Among these, 14 *Wolbachia* strains were detected in 11 host samples, with three samples exhibiting double infections (C51-*Cimex hirundinis*, C12-*Cyanolicimex patagonicus,* and C21-*Psitticimex uritui*). The majority of these genomes ranged in size from approximately 1 to 1.5 Mbp, which aligns well with the sizes reported for complete *Wolbachia* genomes available in the NCBI GenBank. In addition to *Wolbachia*, the genomes of *Symbiopectobacterium, Sodalis, and Serratia* were identified in four, three, and one assembly, respectively. The *Thisiphia* genome, detected in C1-*Afrocimex constrictus*, was fragmented, with very low coverage (1.7–8.6) and BUSCO completeness (Table S3). It was, therefore, not included in the subsequent analyses. The *Symbiopectobacterium* and *Sodalis* genomes were larger than those of *Wolbachia*, ranging between approximately 2 and 3 Mbp. Assembly quality varied substantially among samples, from a complete, closed genome (*Wolbachia* C44-*Paracimex* sp.) to draft assemblies comprising hundreds of scaffolds. Genome completeness, assessed by BUSCO via the ricketssia_db12 database, was high for most of the *Wolbachia* genomes, exceeding 90% in all but two samples (Table S3). For *Sodalis* and *Symbiopectobacterium*, completeness values fell below 50% in some samples when assessed with the pectobacteriaceae_db12 database but increased substantially (exceeding 80%) when evaluated with the enterobacteriales_db12 and bacteria_db12 reference data sets. The GC content varied greatly among the genomes, ranging from 30% to 60%. Genome size and GC content were largely correlated, with a distinct separation between *Wolbachia* (lowest GC and shortest genomes) and the other symbionts.

**TABLE 1:** Main characteristics of the symbionts' genomes^*a*^

Symbiont	Sample	No. of contigs	Genome size (bp)	GC (%)	CDS	Genes	Hypothetical proteins	Coding density (%)	Transposase	Phage regions*	Phage CDS*	Pseudo-genes
*Symbiopecto- bacterium*	C19-*Cacodmus villosus*	110	2,479,358	51.40	3,529	3,575	1,273	79.07	112	4	49	1,457
	C28-*Cacodmus vicinus*	32	2,626,003	50.50	4,264	4,310	1,983	71.22	113	5	57	2,065
	C41-*Leptocimex duplicatus*	35	1,458,153	47.90	1,381	1,426	586	59.2	0	0	0	444
	C57-*Cimex lectulariusH*	414	3,495,616	53.00	3,896	3,954	1,391	84.3	9	6	147	812
	C61-*Cimex hemipterus*	177	1,978,513	49.30	2,684	2,723	1,295	62.61	0	1	9	1,458
*Sodalis*	C5-*Primicimex cavernis*	525	3,378,290	57.10	3,542	3,545	1,262	81.33	15	4	31	580
	C12-*Cyanolicimex patagonicus*	128	2,668,615	56.10	4,294	4,301	2,249	80.47	96	21	366	1,590
	C21-*Psitticimex uritui*	10	1,990,029	39.60	1,244	1,250	612	37.7	0	0	0	275
*Wolbachia*	C1-*Afrocimex constrictus*	220	1,400,906	32.70	2,045	2,048	1,372	68.76	41	1	6	397
	C5-*Primicimex cavernis*	232	1,381,521	35.20	1,105	1,109	559	71.78	14	0	0	138
	**C12-** ***Cyanolicimex patagonicus***	474	2,392,825	34.50	2,363	2,365	1,221	81.32	33	0	0	181
	**C21-** ***Psitticimex uritui***	396	2,146,373	35.90	2,013	2,018	935	79.28	19	0	0	181
	C44-*Paracimex sp*	1	1,136,253	29.50	606	609	138	54.27	0	0	0	130
	C49-*Cimex vicarius*	26	1,219,429	35.20	1,442	1,445	859	79.06	8	0	0	181
	**C51-** ***Cimex hirundinis***	227	2,551,177	35.10	2,631	2,637	1,466	81.23	34	4	45	145
	C56-*Cimex lectulariusB*	125	1,224,977	36.10	1,192	1,195	623	77.75	2	0	0	125
	C57-*Cimex lectulariusH*	123	1,228,894	36.10	1,103	1,107	538	75.99	4	0	0	139
	C61-*Cimex hemipterus*	5	1,147,903	34.00	1,079	1,082	559	63.46	1	0	0	146
	C66-*Cimex adjunctus*	17	1,229,748	35.80	1,368	1,371	774	80.68	15	0	0	170
*Serratia*	C44-*Paracimex sp*	148	4,290,465	59.90	4,091	4,098	1,092	87.89	5	1	40	305

^*a*^ Genomes shown in bold represent double infections with two different strains (see Materials and Methods for details). An asterisk (*) indicates predictions generated by Phastest https://phast. Indexes H and B for *C. lectularius* indicate specimens from human and bat, respectively. Accession numbers of the genome drafts are listed in Table S4.

In addition to standard bacterial coding sequences (CDSs), the genomes present a diverse array of mobile genetic elements (MGEs), notably transposases and phage-related sequences. An intriguing pattern was observed among the five genomes of *Symbiopectobacterium*. Two strains (C28-*Cacodmus vicinus* and C19-*Cacodmus villosus*) presented the greatest number of transposases. A comparison with the large-genome *Symbiopectobacterium purcellii* (strain SyEd1, NZ_CP081864.1) suggested that genome reduction in these two strains is due primarily to the loss of entire genomic regions, often adjacent to transposase elements (Fig. S1). Given the limited number of *Symbiopectobacterium* genomes available for comparison, any interpretation remains speculative; however, a plausible evolutionary scenario can be proposed. The largest genome (C57-*C. lectularius*) harbors relatively few transposases, potentially representing an early stage of symbiont adaptation. Subsequent stages involve the acquisition of numerous transposases, facilitating the deletion of nonessential genomic regions, as evidenced by the intermediate genomes (C28-*Cacodmus vicinus* and C19-*Cacodmus villosus*). This phase of rapid genome degeneration is also characterized by an elevated number of pseudogenes. In the advanced stage, genomes not only become more compact but also exhibit a significant reduction in transposase genes, as observed in the two smallest genomes (C61-*C. hemipterus*; C41-*Leptocimex duplicatus*; Fig. S1).

### Phylogenetic relationships and coevolutionary patterns

#### Cimicidae phylogeny

**Fig 2:**
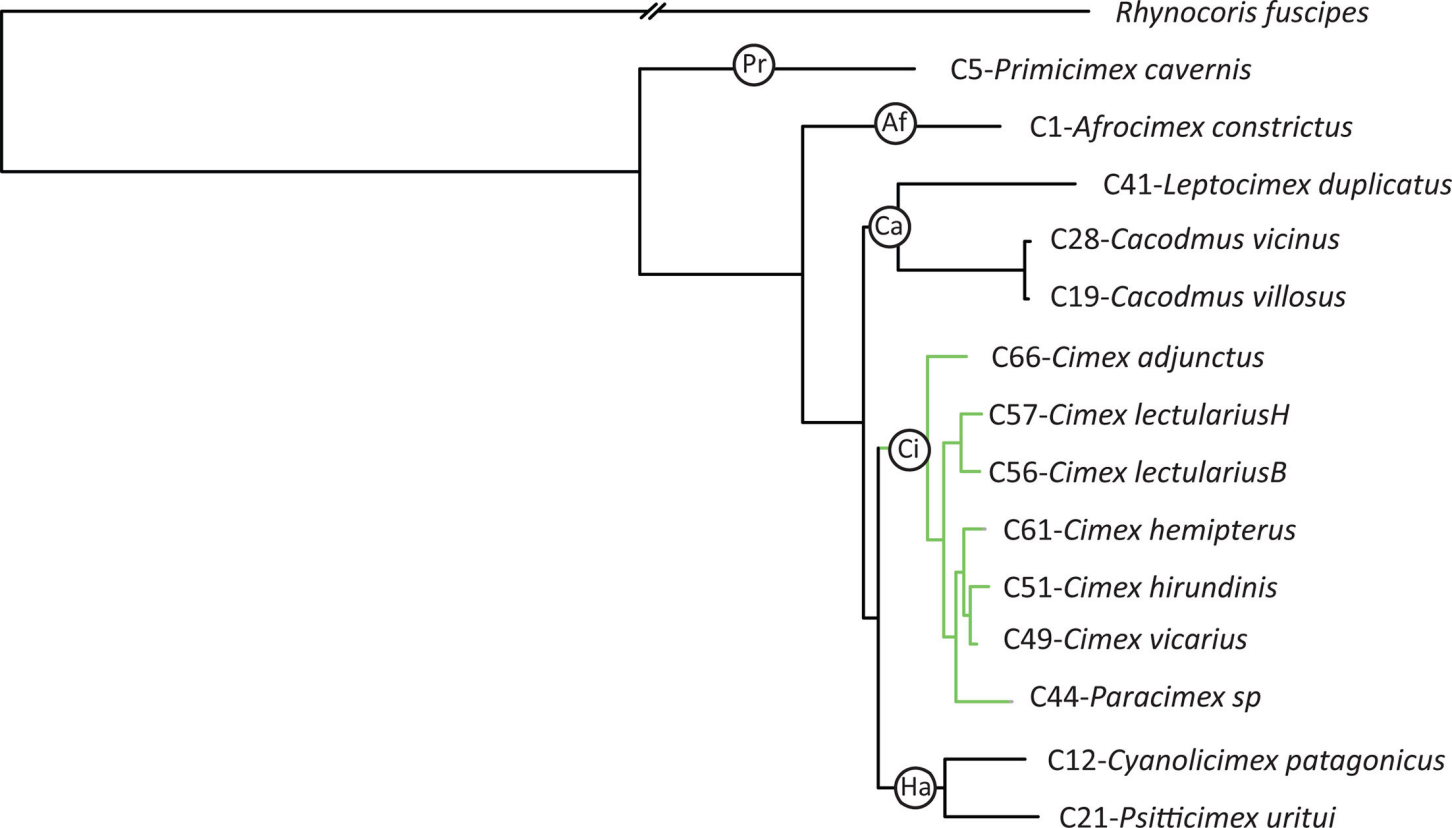
Phylogenetic relationships among Cimicidae samples inferred by ML from an alignment of 35,995 amino acid residues derived from nuclear-encoded proteins. All bootstrap support values were 100. BI recovered an identical topology, with all posterior equal to 1.0. Af = Afrocimicinae, Ca = Cacodminae, Ci = Cimicinae, Ha = Haematosiphoninae, and Pr = Primicimicinae. Green branches indicate the Cimicinae-*Wolbachia* cospeciation.

**Fig 3:**
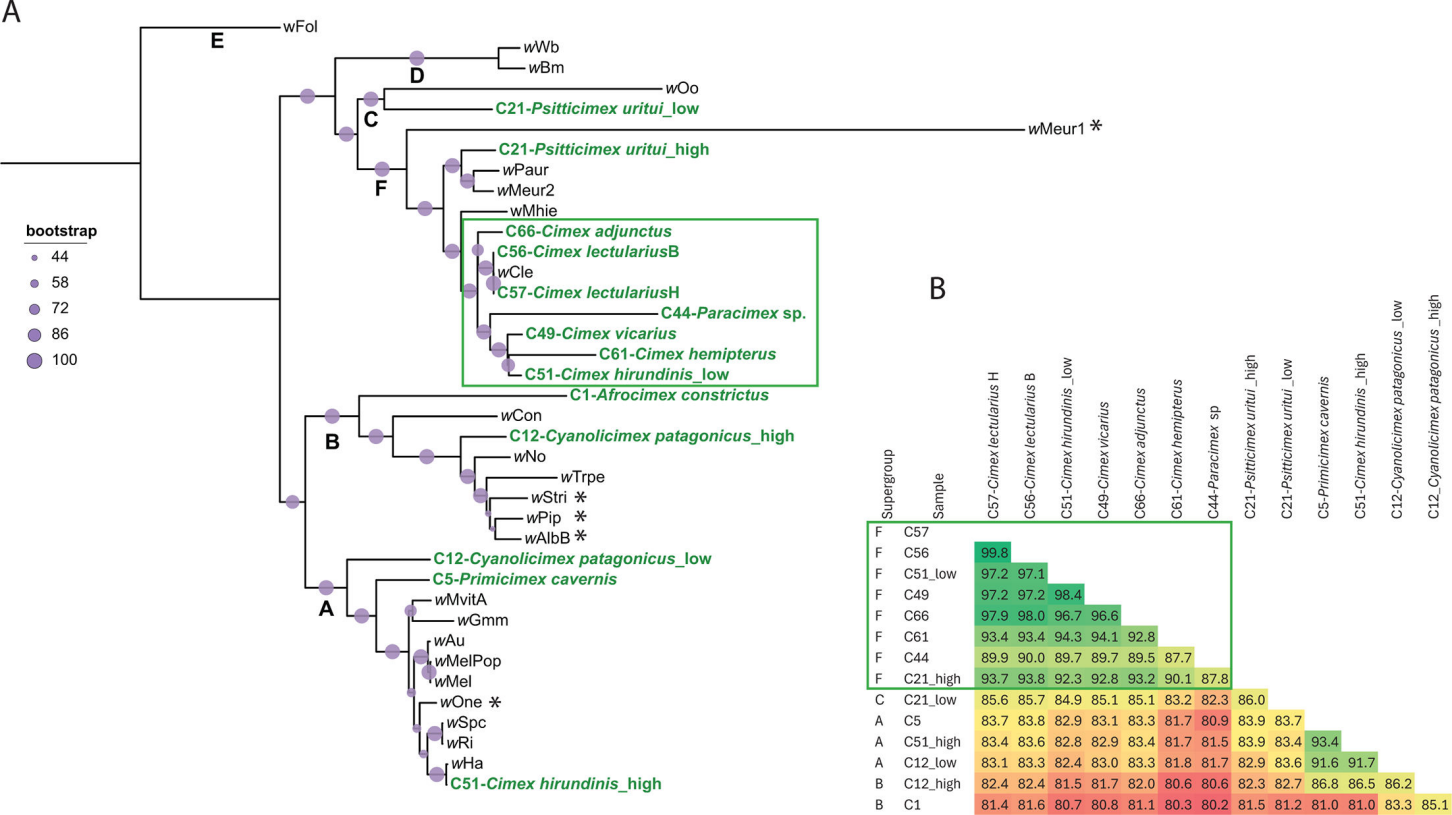
Phylogenetic relationships and genomic similarities among *Wolbachia* strains. (**A**) ML phylogenetic tree inferred from the concatenated matrix of 115 single-copy orthologs (27,850 amino acid residues). Letters at the nodes indicate *Wolbachia* supergroups. Strains identified in this study from cimicid species are highlighted in bold green. The designations “high” and “low” indicate differences in sequencing coverage between co-occurring strains in the same host. The green rectangle highlights the coevolving lineage. Asterisks mark branches whose positions differ in BI analysis. (**B**) Average nucleotide identity (ANI) of *Wolbachia* strains associated with cimicid hosts. The green rectangle highlights the coevolving lineage. Accession numbers for all included taxa are provided in Table S4.

Both maximum likelihood (ML) and Bayesian inference (BI) phylogenetic analyses, which are based on an alignment of 35,995 amino acid residues derived from nuclear-encoded proteins, yielded identical topologies with strong support (all posterior probabilities of 1.0 and bootstrap values of 100; Fig. 2). This topology differs in two key aspects from the phylogeny published by Roth et al. (26), which was based on four genes. First, Cacodminae and Haematosiphoninae form a paraphyletic group relative to the monophyletic Cimicinae. Second, within Cimicinae, *Paracimex* branches within *Cimex*, alternating the internal topology of the subfamily. Notably, this revised topology is not only well supported but also congruent with the phylogeny of the corresponding *Wolbachia* strains (highlighted in green; compare to Fig. 3).

### Symbiont phylogenies

#### Wolbachia

Phylogenetic analyses via ML and BI based on 115 single-copy orthologs (27,850 amino acid residues) recovered largely identical topologies, with only a few minor differences (Fig. 3; Fig. S2). These analyses revealed substantial phylogenetic diversity among the *Wolbachia* strains from cimicid hosts. Strains belonging to supergroup F, associated with Cimicinae species (including *Paracimex*), formed a well-supported monophyletic cluster whose topology mirrored that of their host, as recovered in this study (Fig. 2), which is consistent with a previously proposed coevolutionary scenario (19). In contrast, the remaining seven strains, affiliated with other supergroups, clustered with strains from unrelated insect hosts and showed no correspondence with cimicid phylogeny. In four samples exhibiting double infections, the co-occurring *Wolbachia* strains occupied distinct positions in the phylogenetic tree, indicating divergent evolutionary origins. Genome-wide ANI analyses supported these patterns: within the coevolving supergroup F cluster, ANI values exceeded 90% (with the exception of the *Paracimex*-associated strain), whereas ANI values between co-occurring strains from the same host ranged from 82% to 86%, confirming their distinct phylogenetic affiliations.

#### Symbiopectobacterium

**Fig 4:**
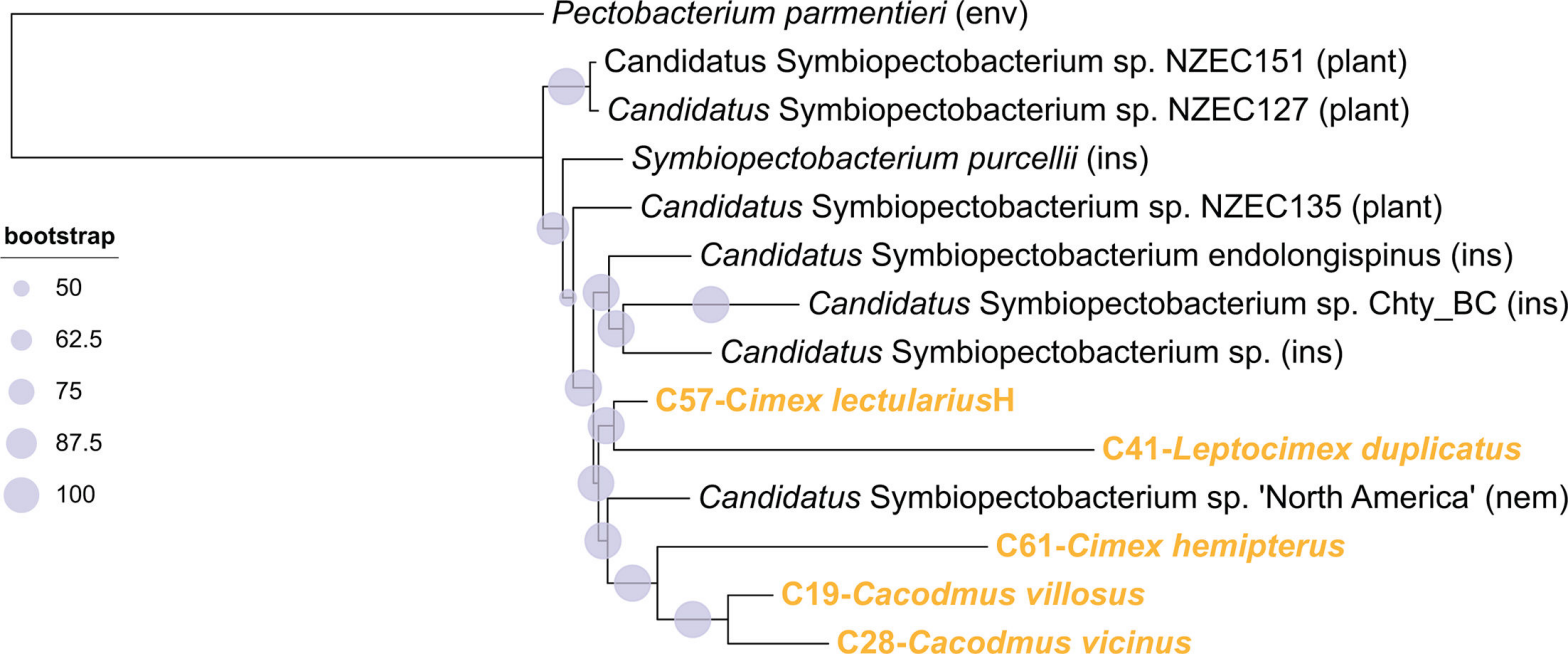
Phylogenetic relationships of the *Symbiopectobacterium* strains inferred by ML analysis of 340 single-copy orthologs (90,300 amino acid residues). Strains identified in this study from cimicid species are shown in bold orange. The BI analysis recovered an identical topology, with all posterior probabilities equal to 1. Abbreviations following the strain names indicate the source of the sample: environmental (env), insects other than Cimicidae (ins), nematode (nem), and plant. Accession numbers and details of all included taxa are provided in Table S4.

Both ML and BI analyses of five *Symbiopectobacterium* strains, which were based on 340 single-copy orthologs (totaling 90,300 amino acid residues), revealed an identical topology that clustered all five cimicid-associated strains together with a nematode-associated strain (*Candidatus* Symbiopectobacterium sp. “North America”) (Fig. 4). Despite their apparent close phylogenetic relationships, the positions of the cimicid-associated strains suggest at least four independent acquisitions of *Symbiopectobacterium* by bedbugs. Only in one instance (a pair of sister *Symbiopectobacterium* strains, C28 *C. vicinus* and C19 *C. villosus*) did the symbiont phylogeny correspond with the host relationship as both were isolated from closely related *Cacodmus* species. The remaining three strains clustered without any apparent correspondence to their host phylogeny. Although both the ML and BI analyses revealed the same topology with well-supported nodes, notably, at least two strains (C41- *Leptocimex duplicatus* and C61- *Cimex hemipterus*) formed long branches, which could result in topological artifacts.

#### Sodalis

**Fig 5:**
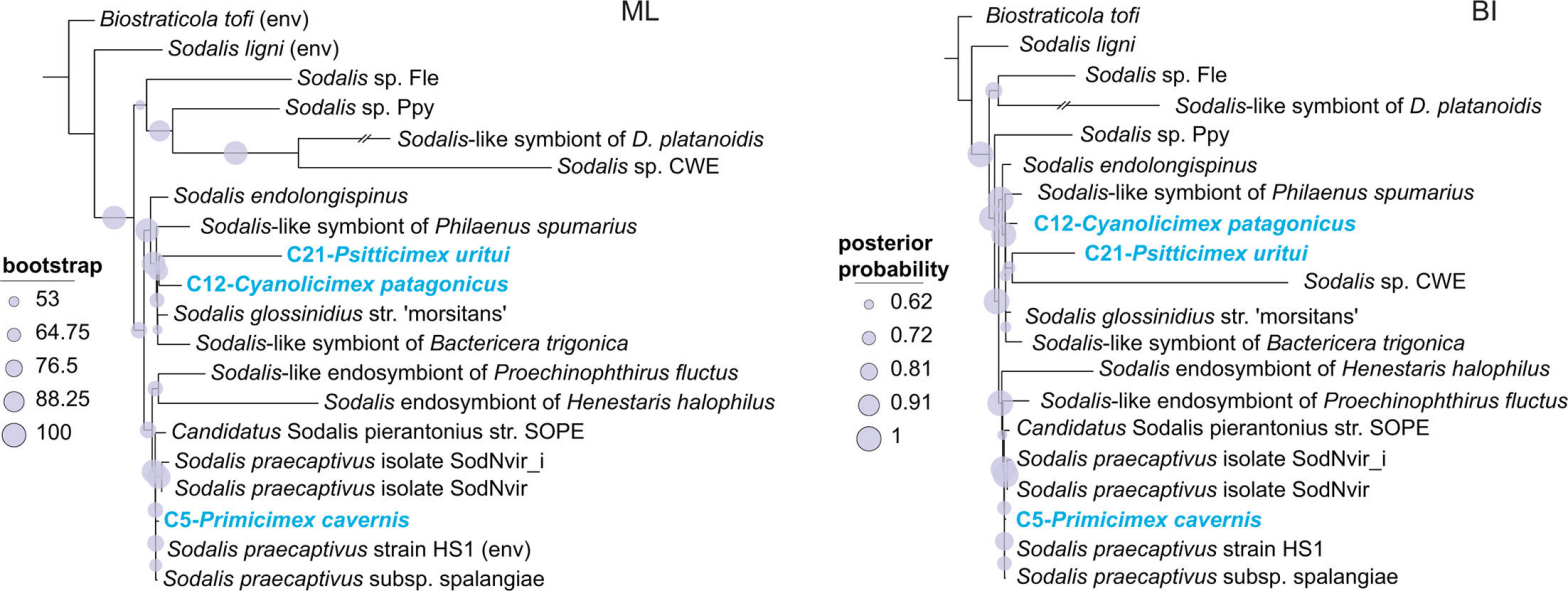
Phylogenetic relationships of the *Sodalis* strains inferred by ML and BI analyses of 41 single-copy orthologs (11,296 amino acids). Strains obtained in this study from cimicid species are shown in bold blue. All other strains, except three environmental strains (labeled “env”), were isolated from insects other than Cimicidae. Accession numbers and details of all included taxa are provided in Table S4.

The phylogenies inferred by ML and BI analyses based on 41 single-copy orthologs (11,296 amino acids) differed in several aspects (Fig. 5), but both consistently placed the bedbug-associated *Sodalis* strains into two distinct clusters: the first comprising C12-*Cyanolicimex patagonicus* and C21-*Psitticimex uritui* and the second comprising C5- *Primicimex cavernis*. This well-supported topology suggests at least two independent origins of Cimicidae*–Sodalis* symbiosis. Furthermore, the phylogenetic affiliations and branch lengths within these two clusters indicated different characteristics of the symbiotic associations. The C12-*Cyanolicimex patagonicus* and C21-*Psitticimex uritui* strains are related to other insect symbionts and possess relatively long branches (particularly the latter), in contrast to C5-*Primicimex cavernis*, which is located on a very short branch among the strains of *S. praecaptivus*. Evolutionary interpretation of the Cimicidae*–Sodalis* association is, however, complicated by an inconsistent position of the two long-branched strains associated with the Haematosiphoninae species. While the ML analysis positioned them as sister taxa, the BI tree placed them in a paraphyletic arrangement, where the C21-*Psitticimex uritui* strain was related to another particularly long-branch bacterium, the *Sodalis* CWE strain described from a chewing louse (27). Both of these alternative placements were supported only by short branches and low bootstrap and posterior probability values, indicating potential instability in these relationships.

#### Serratia

**Fig 6:**
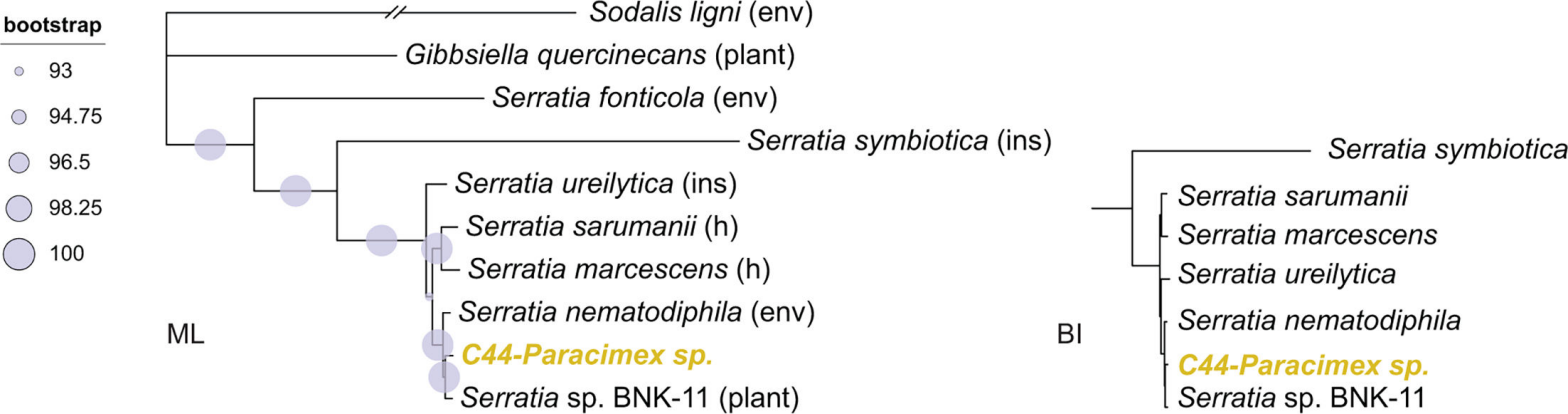
Phylogenetic placement of the *Serratia* symbiont associated with *Paracimex* sp. Based on 1,271 single-copy orthologs (394,084 amino acids), both ML and BI analyses inferred identical topologies, with only minor differences observed within the cluster of the short-branched *Serratia* strains. The strain obtained in this study is shown in bold yellow. Abbreviations following the strain names indicate the source of the sample: environmental (env), insects other than Cimicidae (ins), human (H), and plant. Accession numbers and details of all included taxa are provided in Table S4.

Both analyses, which were based on 1,271 single-copy orthologs (394,084 amino acids), consistently placed the sole identified *Serratia* strain (C44- *Paracimex* sp.) in a clade with the nematode symbiont *Serratia nematodiphila*, a nematode-associated bacterium, and *Serratia* sp. strain BNK-11, which is of unclear origin (Fig. 6). This grouping was distinct from the clade containing the aphid symbiont *S. symbiotica*.

### Metabolic capacity

**Fig 7:**
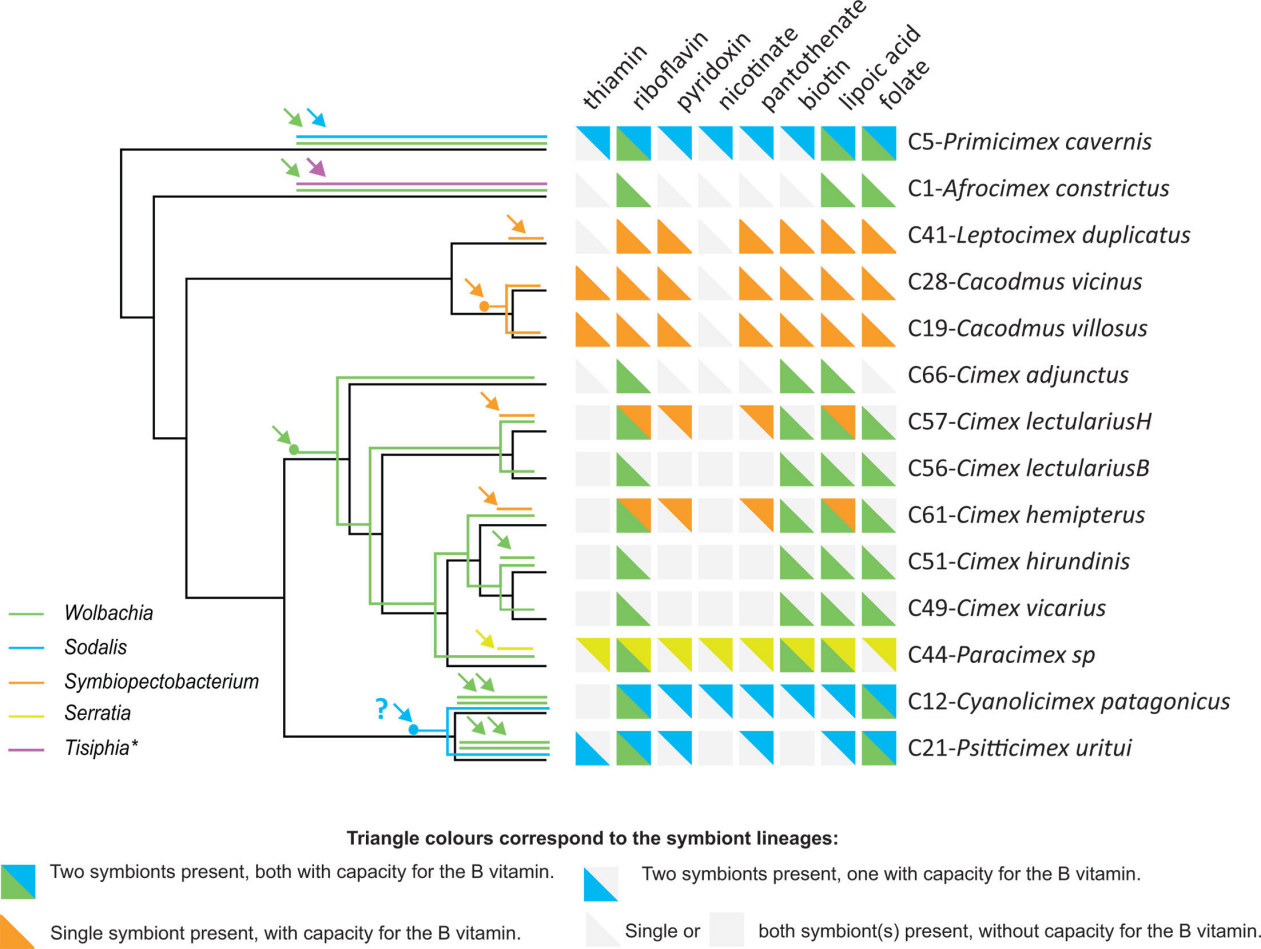
Origins of symbionts and their metabolic capacities for B vitamins mapped onto the phylogenetic relationships of their hosts. Arrows indicate hypothetical independent origins. Dots represent potential cospeciation events between hosts and symbionts (the question mark in the *Sodalis* lineage highlights uncertainty regarding cospeciation versus two independent origins). The asterisk at *Tisiphia* denotes that this bacterium was excluded from the metabolic overview due to low genomic coverage.

A comparison of metabolic capacities across the analyzed symbionts (overview in Table S5) revealed a pattern shaped by two main factors. First, in many metabolic pathways, the presence or absence of genes is influenced by the symbiont’s phylogenetic position. This is particularly evident when two phylogenetically distant groups, *Wolbachia* (Alphaproteobacteria) and *Sodalis/Symbiopectobacterium* (Gammaproteobacteria), are compared. Notable examples include several amino acid biosynthesis pathways, ABC transporters, two-component systems, and lipopolysaccharide synthesis (Table S5). Second, metabolic capacity is determined by the extent of genome degradation. This is clearly illustrated by the *Symbiopectobacterium* strains C41-*Leptocimex duplicatus* and C61-*Cimex hemipterus,* which exhibit considerably fewer complete metabolic pathways than the other *Symbiopectobacterium* strains (reflected in the smaller size of the two genomes) (Table 1, Fig. 7). This reduction appears to reflect genuine genomic degradation rather than incomplete assemblies, as evidenced by the lower GC content and long branches these strains form in phylogenetic trees, both of which are indicative of ongoing genomic degeneration.

A detailed assessment of vitamin B synthesis (Fig. 7) revealed that only two pathways, riboflavin and lipoic acid, were universally functional in all symbionts, including different symbiont genera inhabiting a single host. The significance of the riboflavin pathway deduced from its functionality in all the samples agrees with the results of several previous studies (e.g., 28–30). In a comparative genomic study of *Wolbachia* strains, the synthesis pathway for riboflavin was the only synthetic pathway of vitamins B conserved among diverse insects, and its importance for bed bug growth, survival, and reproduction was demonstrated experimentally (18). Similarly, riboflavin synthesis has been found to be universally functional in all 11 analyzed strains of another symbiotic bacterium, the genus *Arsenophonus,* associated with hippoboscids (31). Among the 22 symbionts analyzed in our study (with *Wolbachia* double infections treated as a single strain for metabolic purposes), 13 lacked the gene annotated for the dephosphorylation step in the standard riboflavin pathway, as defined in the KEGG database. However, this step can be catalyzed by several alternative enzymes, and similar “gaps” have been reported in other symbiotic bacteria (32). Given the retention of all other genes in the pathway, this finding suggests that riboflavin synthesis remains functional, despite the absence of a canonical dephosphorylation gene. A third pathway, folate biosynthesis, was found to be functional in most symbionts. The two exceptions were the *Wolbachia* strains from *Paracimex* sp. and *C. adjunctus*. In the case of *Paracimex* sp., the co-occurring *Serratia* symbiont retained all the functional B vitamin pathways, leaving the *C. adjunctus* sample the only one without apparent capacity for folate synthesis.

Three B-vitamin biosynthesis pathways (thiamine, pyridoxal, and pantothenate) were nonfunctional in all *Wolbachia* strains but were largely retained in the other symbionts. In particular, the pyridoxal and pantothenate pathways seem to be functional in all strains of *Symbiopectobacterium*, *Sodalis,* and *Serratia*. In the C57-*C. lectularius* sample, thiamine synthesis may be potentially functional if the partial biosynthetic capacities of the two coresident symbionts, *Wolbachia* and *Symbiopectobacterium*, are combined. In contrast, the capacity for nicotinate and cobalamin biosynthesis was absent in most or all symbionts. The most intriguing pattern was observed in biotin synthesis. Biotin is commonly considered essential for obligate blood-feeding insects and is supplemented by symbionts (5). This seems to be supported by independent acquisition of the biotin operon by horizontal gene transfer (HGT) in several symbiotic bacteria (11, 33). Furthermore, an experimental study demonstrated that supplementation with biotin and riboflavin effectively restored the fitness of bed bugs following the elimination of their symbionts (18). Our data revealed that the biotin pathway was functional in most, but not all, of the analyzed symbionts. One line of evidence highlighting the potential importance of biotin supplementation is its distribution in *Symbiopectobacterium* strains. The pathway was present in strains from all three Cacodminae species, where *Symbiopectobacterium* is the sole symbiont, but absent in both strains from Cimicinae species, where biotin is instead produced by co-occurring *Wolbachia*. Moreover, the genome of the *Wolbachia* strain C44-*Paracimex* carries a duplicated biotin operon, with the two copies complementing each other: in one copy, the bioB gene is disrupted by a premature stop codon, whereas the other copy contains functional bioB and disrupted BioF, bioD, and bioA (Fig. S3). The signs of gene degradation within this pathway in some other *Wolbachia* strains further complicate the interpretation of the essentiality of biotin. Although these strains retain the biotin operon (originally obtained by HGT), several genes appear to be truncated or fragmented due to premature stop codons and were not annotated as functional by BlastKoala. In at least two samples (C1-*Afrocimex constrictus* and C21-*Psitticimex uritui*), the biotin pathways of their symbionts are very likely nonfunctional.

### Symbiont origins and coevolutionary history

An overview of the 14 Cimicidae samples revealed that their associations with symbiotic bacteria have undergone remarkably dynamic evolution, with multiple independent symbiont acquisitions and frequent co-occurrences of multiple symbionts within individual hosts (Fig. 7). Interestingly, *Symbiopectobacterium*, previously known as a facultative symbiont that co-occurs with obligate *Wolbachia*, was detected in this study as the sole symbiont in three Cacodminae species, which lack *Wolbachia*. Mapping the symbiont associations onto the cimicid phylogeny suggested at least 16 independent origins (Fig. 7). Only three of these origins show evidence of subsequent coevolution across host species boundaries: seven strains of *Wolbachia* associated with Cimicinae, two *Symbiopectobacterium* strains from the two *Cacodmus* species, and two *Sodalis* strains from the Haematosiphoninae species. The first two cases are straightforward: the symbionts form well-supported monophyletic lineages, each associated with a group of closely related hosts. The pattern observed in *Sodalis*, however, is less clear. The two host genera (*Cyanolimex* and *Psitticimex*) belong to the same subfamily but are not sister taxa or particularly closely related genera. If the observed pattern reflects a process of host-symbiont cospeciation, the full coevolutionary history of this *Sodalis* lineage likely involves additional genera/species within Haematosiphoninae and their associated *Sodalis* strains. This interpretation is also consistent with the long branches of both coevolving *Sodalis* strains in our phylogenetic analysis (C12-*Cyanolicimex patagonicus* and C21-*Psitticimex uritui*).

Considering the high number of symbiont acquisitions, it is noteworthy that they involve only a limited set of bacterial taxa. The majority are represented by different strains of *Wolbachia*, with eight independent origins, followed by four origins from *Symbiopectobacterium*, two from *Sodalis*, and one from *Serratia* and *Tisiphia*. The distribution, coevolutionary patterns, and genome characteristics of these bacteria suggest that the different strains have reached various stages in their symbiogenesis. These stages are reflected in traits such as genome reduction, metabolic degradation, shifts in nucleotide composition, and the presence of mobile elements.

The observed patterns also raise several important questions for future research. One key direction is to better understand the overall diversity of symbionts across Cimicidae, which will require addressing phylogenetic gaps in host sampling. The importance of this step is underscored by the identification of *Symbiopectobacterium* as the sole symbiont in Cacodminae species, extending the known obligate symbionts in Cimicidae to at least two genera, *Wolbachia* and *Symbiopectobacterium*. The data set analyzed in this study covers all currently recognized subfamilies of bed bugs, except for Latrocimicinae (a group consisting of a single bat-related species). Within the best-represented subfamily, Cimicinae (with six included species), our sample demonstrated two patterns: (i) *Wolbachia*–Cimicinae cospeciation across six host species and (ii) several independent origins of *Wolbachia, Symbiopectobacterium*, and *Sodalis* strains. However, more comprehensive sampling will be essential to gain deeper insights into the symbiotic associations in other subfamilies. For example, Haematosiphoninae, characterized by broad morphological, ecological, and host diversity across the Americas, is represented here by only two *Sodalis*-harboring species. Expanding sampling in this group could clarify the coevolutionary history of *Sodalis* and potentially uncover novel symbiotic associations.

Similarly, deeper insights into the relationship between Cacodminae and *Symbiopectobacterium* may be achieved by including additional taxa, such as *Aphrania* (a genus related to *Cacodmus*) and the species-rich genera *Stricticimex* and *Loxaspis*, both of which are related to *Leptocimex*. An especially intriguing direction is to extend analyses to Polyctenidae, a group recently proposed to have evolved within Cimicidae (34). Polyctenids are more specialized than cimicids and spend their entire life cycle on bat hosts (an adaptation supported by vivipary). Comparison of cimicids and polyctenids may shed light on how ecology and specialization influence the dynamics of symbiont acquisition and the stability of symbiont associations. Another important goal is to conduct population-level screenings to determine which of the detected strains are fixed in their host species. Given the primary nutritional role of symbionts, identifying which compounds are truly essential for cimicid hosts, particularly B vitamins, is especially important. In this study, several consistent patterns emerged, such as the universal production of riboflavin and the widespread degeneration of the thiamine biosynthesis pathway. A similar pattern has been reported across several *Arsenophonus* strains associated with obligate blood-feeding flies in the family Hippoboscidae (31). On the other hand, inconsistencies in other vitamins are notable and warrant further investigation. A particularly illustrative case is biotin. Multiple lines of evidence suggest that biotin synthesis is essential for symbiont function (e.g., the repeated acquisition of the biosynthetic pathway via horizontal transfer in symbiotic bacteria or the pathway duplication observed in this study). However, this is difficult to reconcile with the apparent nonfunctionality of the biotin pathway in some symbionts. This discrepancy may reflect limitations in our ability to assess pathway functionality with confidence, or it may indicate different nutritional requirements among host species. Resolving these questions will likely require complementary transcriptomic and experimental approaches beyond genomic data alone.

## MATERIALS AND METHODS

### Samples and DNA preparation

Cimicid samples were collected from various localities across four continents between 2005 and 2015 (Table S1). For 16S rRNA amplicon analysis, DNA was extracted from 35 individuals via the E.Z.N.A. Insect DNA Kit (Omega BIO-TEK). On the basis of amplicon profiles, DNA concentrations, and integrity, 14 samples representing 13 different species (*C. lectularius* was represented by two samples, one from a human host and the other from a bat) were selected for metagenomic analysis (Table S4). DNA concentrations were measured via a Qubit 2.0 fluorometer (Invitrogen, Carlsbad, CA, USA), and integrity was assessed via agarose gel electrophoresis. To enrich bacterial DNA, host methylated DNA was selectively removed via the NEBNext Microbiome DNA Enrichment Kit (New England BioLabs). The final DNA concentrations were again quantified via a Qubit 2.0 fluorometer with high-sensitivity reagents.

### Amplicons

Using the QIAGEN Multiplex PCR Kit (Qiagen, Hilden, Germany), we amplified the V3–V4 region of the 16S rRNA gene from all samples, as well as from four blank (negative) PCR controls and two commercial gDNA (positive) controls (ATCC MSA-1000 and MSA-1001, each containing the same ten bacterial species in different proportions). Two-step PCR was performed with the 341F and 805R primers, which contained a staggered spacer and Illumina overhang adapters, followed by index PCR to add sample‐specific barcodes (Illumina 16S Metagenomic Sequencing Library Preparation Guide). The purified amplicons were quantified, pooled equimolarly, and sequenced on an Illumina MiSeq via v2 chemistry (2 × 250 bp paired-end reads).

The raw Fastq files were processed via USEARCH v11.0.667 (35), including primer stripping, read merging, trimming, quality filtering, and OTU picking. The reads were merged, allowing for zero mismatches within the alignment. The merged reads were quality-filtered with the stringent option fastq_maxee, which was set to 0.5, and trimmed to 400 bp. The OTU table was generated by clustering these sequences at 100% identity, followed by *de novo* OTU picking via USEARCH global alignment at 97% identity, including chimera removal (35). Taxonomic assignment was conducted via BLASTn against the SILVA_138.2 database (https://www.arb-silva.de/no_cache/download/archive/release_138_2/Exports/). Data filtering, rarefaction, and heatmap visualization were carried out in R via microeco v0.16.0 (36) and ggplot2 v3.4.2 (37). In detail, the OTU table was taxonomically filtered to remove archaeal, eukaryotic, mitochondrial, and chloroplast OTUs. We expected symbiont OTUs to be abundant, so any OTU comprising <1% of reads in a sample was set to zero for that sample (presence across other samples was not considered). Finally, the data set was rarefied to 1,000 reads per sample, and samples with fewer reads were excluded from further analysis.

### Metagenomics

The shotgun genomic libraries were prepared from enriched gDNAs, multiplexed, and sequenced on a NovaSeq 6000 from both ends. Metagenomic raw reads were quality-trimmed via Trimmomatic (38) with the -phred33 option and assembled with SPAdes (39) via the parameters --meta -t 50 k 21,33,55,77,99,127. For several samples, the assembly failed due to memory allocation issues, likely caused by the high complexity of the data. In these cases, the data were downsampled to 50% using seqtk (https://github.com/lh3/seqtk). From each successful assembly, a BLAST database was created and screened for contigs corresponding to symbionts indicated by the amplicon data: *Wolbachia*, *Symbiopectobacterium*, *Sodalis*, *Serratia,* and *Tisiphia*. To obtain representative queries, we downloaded genomes from NCBI that covered the diversity of each of these five bacterial taxa (Table S4) and used the Roary program (40) to construct pangenomes in which each gene was represented only once. These data sets were used as BLAST queries against each assembly-derived database. Contigs retrieved by these queries were annotated via PROKKA (41), and their taxonomic origins were validated via a “back-BLAST” procedure: all annotations from each contig were extracted and compared against the NCBI nr database. For each contig, the bacterial genus with the greatest number of hits was considered the contig’s taxonomic origin; contigs with predominant eukaryotic hits were excluded from further analysis. This procedure yielded sets of contigs putatively representing five symbiotic genera: *Wolbachia*, *Sodalis*, *Symbiopectobacterium*, *Serratia,* and *Tisiphia*.

*Double infections*. As predicted by the amplicon results, three samples (C51–*Cimex hirundinis*, C12–*Cyanolicimex patagonicus*, and C21–*Psitticimex uritui*) each harbored two distinct *Wolbachia* strains. Metagenomic data provided two additional lines of evidence. First, the Orthofinder (42) results revealed an excess of duplicated orthologs in these samples, suggesting the presence of more than one *Wolbachia* genome. Second, BLAST searches using annotated *Wolbachia* genes as queries against the complete metagenomic assemblies retrieved most genes in two copies, located on contigs with distinct coverage. Because the two *Wolbachia* genomes within a single host sample could not be fully separated (due to overlapping contig coverage), we employed different strategies depending on the analysis. For phylogenetic analysis, we prioritized selecting genes that reliably represented the two distinct genomes over maximizing the total gene count. To achieve this, the contigs were grouped into “high coverage” and “low coverage” sets, excluding those with intermediate coverage. These two sets were treated as separate genomes in a new Orthofinder run and subsequent phylogenetic analyses. For metabolic analyses, we used the complete set of all *Wolbachia* genes from a sample as a single “composite genome” to ensure the inclusion of all genes potentially involved in the metabolic pathways reconstructed for the sample. ANI among the *Wolbachia* strains was calculated using a web-based ANI calculator (43). The main genomic characteristics (Table 1) were derived mostly from the PROKKA annotations. Additionally, we used PHASTEST (44) as an alternative tool to identify phage-associated sequences and Pseudofinder (45) to quantify pseudogenes. The completeness of the genome draft was assessed with BUSCO v4.0.6 (46).

### Phylogeny

Phylogenetic reconstructions were conducted for five sets: Cimicidae hosts, *Wolbachia*, *Symbiopectobacterium*, *Sodalis*, and *Serratia*. Owing to the enrichment of bacterial sequences at the expense of host DNA (see above), we did not base the Cimicidae phylogeny on genome drafts. Instead, to obtain representative sets of orthologous sequences, we adopted the following strategy: coding sequences (CDSs) were extracted from the reference genome of *Cimex lectularius* (GCF_000648675.2) and used as queries to BLAST the assembly databases of the cimicid samples. Open reading frames (ORFs) in the retrieved contigs were identified via the built-in algorithm Geneious Prime (47). These sequence sets were then processed via Orthofinder to identify orthologous sequences. As an outgroup, we included the reduviid species *Rhynocoris fuscipes* (NCBI accession: GCA_040020575.1), for which ORFs were identified via the same method. Single-copy orthologs were aligned via MAFFT (48) with default settings. The alignments were concatenated, and unreliably aligned regions were removed via GBlocks (49) with default parameters. Phylogenies were inferred via two approaches. First, Bayesian inference (BI) was performed in PhyloBayes MPI (50), running two independent chains under the CAT-GTR model, with the number of generations determined by the convergence parameter (maxdiff <0.1). Second, maximum likelihood (ML) analysis was carried out in IQtree (51), with matrices partitioned by genes. The best partitioning scheme and substitution models were selected automatically, and node support was assessed via 1,000 ultrafast bootstrap replicates. For the four bacterial data sets, phylogenetic reconstructions were based on their annotated genome drafts (bacteria included into the analyses, and accession numbers of their genomes, are listed in Table S4). The entire workflow, from ortholog identification to alignments and phylogenetic analyses, followed the same procedure as described above for the cimicid hosts. The trees were visualized via the program iTOL (52). For *Tisiphia,* with only a fragmentary low-coverage draft genome, we based the phylogenetic placement on the NCBI fast minimum evolution algorithm and used two different genes in independent analyses (Fig. S4).

### Metabolic analysis

Metabolic capacities were assessed for 22 samples (Table S4; *Tisiphia* was not analyzed due to the low genome coverage). In addition to the newly assembled symbiont genomes, two reference strains, *Symbiopectobacterium purcellii* (NZ_CP081864) and *Sodalis praecaptivus* (GCF_039646275.1), were included for comparison. In samples with *Wolbachia* double infections (C51-*Cimex hirundinis,* C12-*Cyanolicimex patagonicus, and* C21-*Psitticimex uritui*), the two genomes were treated as a single entry by combining all *Wolbachia* genes into one set per host (see above). Metabolic functions were evaluated via the KEGG database (53). K numbers linking genes to metabolic functions were assigned to all PROKKA-annotated CDSs via the BlastKOALA server (54), and the resulting metabolic capacities were mapped to KEGG-defined metabolic pathways. Particular emphasis was placed on evaluating the functionality of B-vitamin biosynthetic pathways. Following the approach from our previous work (31), each missing gene was assessed individually. Two criteria were considered: (i) genes absent across all or most symbionts within otherwise intact pathways were assumed to be potentially nonessential; and (ii) pathways lacking a gene known to have alternative enzymes were also considered potentially functional.

## Data Availability

Illumina reads and genome drafts have been deposited in the NCBI under accessions SAMN48908988-SAMN48909022 for amplicons and SAMN48887401-SAMN48887414 for metagenomic data. All accession numbers, including those for genome drafts, are provided in Tables S1 and S4. Matrices used for phylogenetic analyses are available in Mendeley Data under https://doi.org/10.17632/ny4ymjsxtd.1.

## SUPPLEMENTAL MATERIAL

Supplemental Figures msystems.01247-25-s0001.pdf Figures S1 to S4.

Table S1 msystems.01247-25-s0002.xlsx Amplicon metadata.

Table S2 msystems.01247-25-s0003.xlsx Illumina short-read sequencing data.

Table S3 msystems.01247-25-s0004.xlsx BUSCO values for the symbiont genomes.

Table S4 msystems.01247-25-s0005.xlsx Accession numbers.

Table S5 msystems.01247-25-s0006.xlsx Overview of metabolic capacities.

ASM does not own the copyrights to Supplemental Material that may be linked to, or accessed through, an article. The authors have granted ASM a non-exclusive, world-wide license to publish the Supplemental Material files. Please contact the corresponding author directly for reuse.
