# Supplementary material for: Dynamic but constrained: repeated acquisitions of nutritional symbionts in bed bugs (Heteroptera: Cimicidae) from a narrow taxonomic pool
Source: mSystems. 2025 Nov 10;10(12):e01247-25. doi: 10.1128/msystems.01247-25 (PMC12710357; doi:10.1128/msystems.01247-25)
Supplement: Supplemental Figures — Figures S1 to S4. [file msystems.01247-25-s0001.pdf]

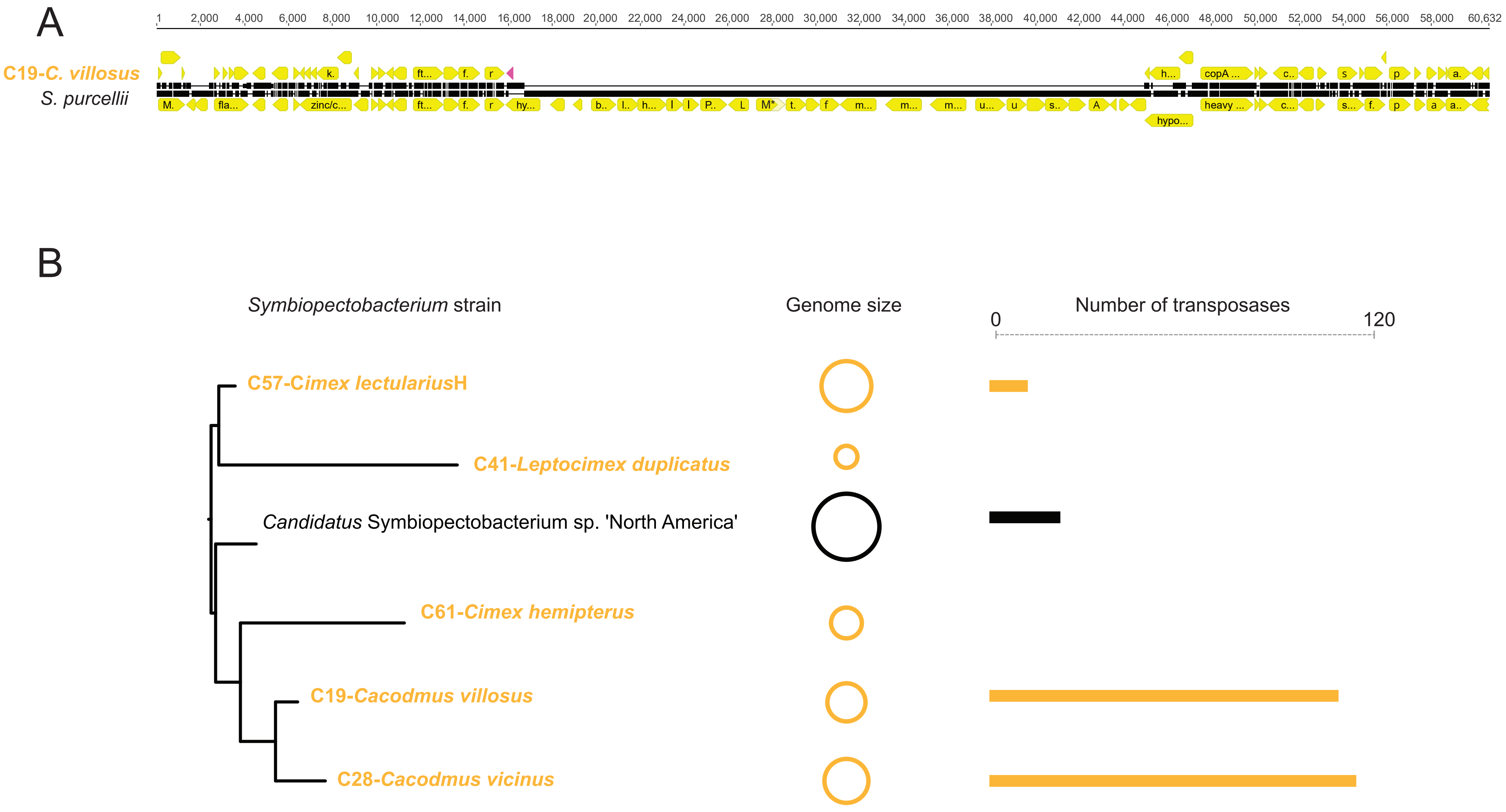

Supplementary figure S1. A: An example of gene loss in C19-*Cacodmus villosus* genome compared to *Symbiopectobacterium purcelii*. Yellow shapes=CDSs, pink triangle=transposase. B: Comparison of genome sizes and transposase numbers among the *Symbiopectobacterium* strains. Detailed genome characteristics are provided in Table 1.

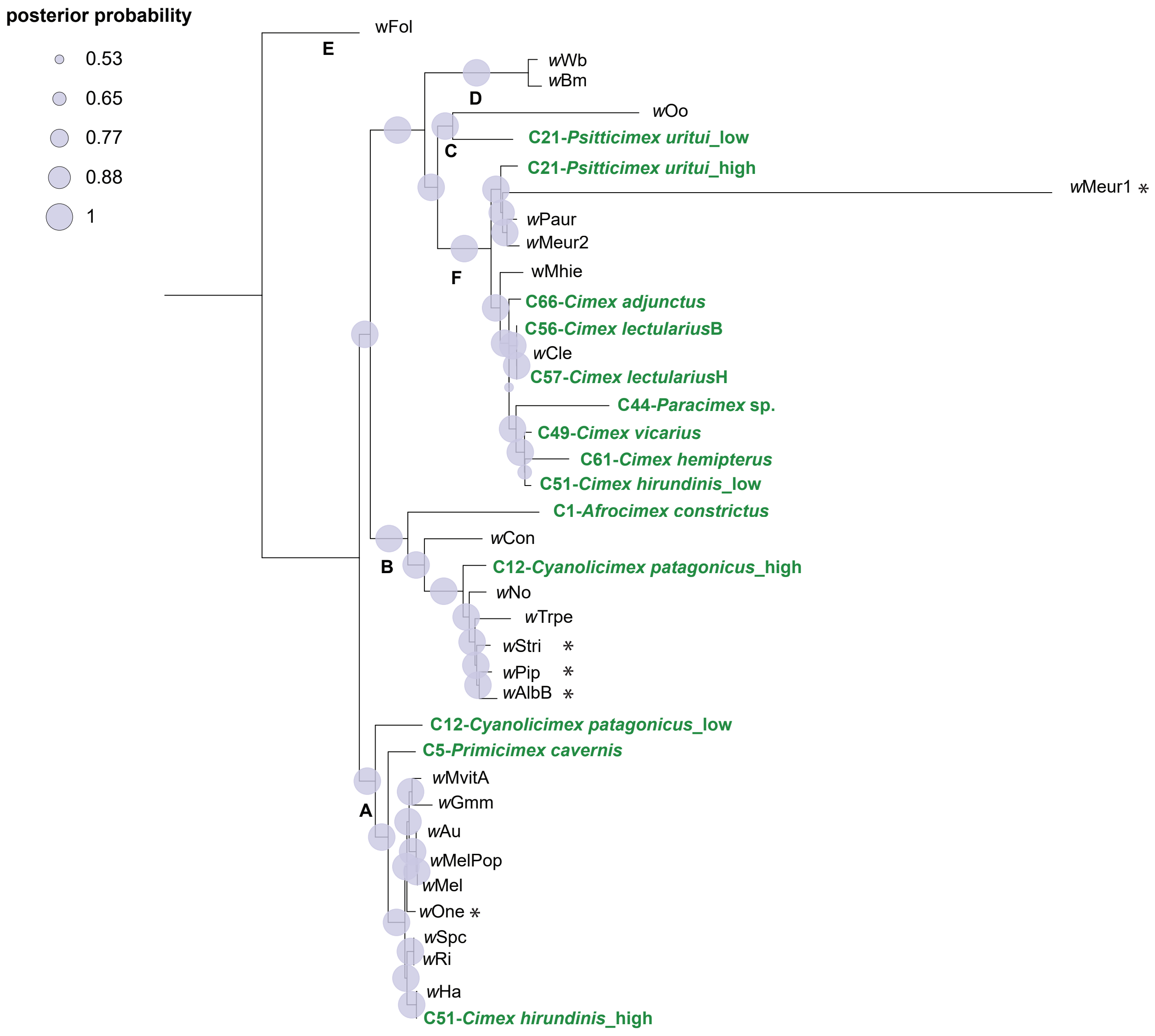

Supplementary figure S2. Phylogenetic relationships inferred by BI from concatenated matrix of 115 single-copy orthologs (27,850 amino acid residues). Letters at the nodes indicate *Wolbachia* supergroups. Strains identified in this study from cimicid species are highlighted in bold green. The designations "high" and "low" indicate differences in sequencing coverage between co-occurring strains in the same host. Asterisks mark branches whose positions differ in ML analysis. Accession numbers for all included taxa are provided in Supplementary Table S4.

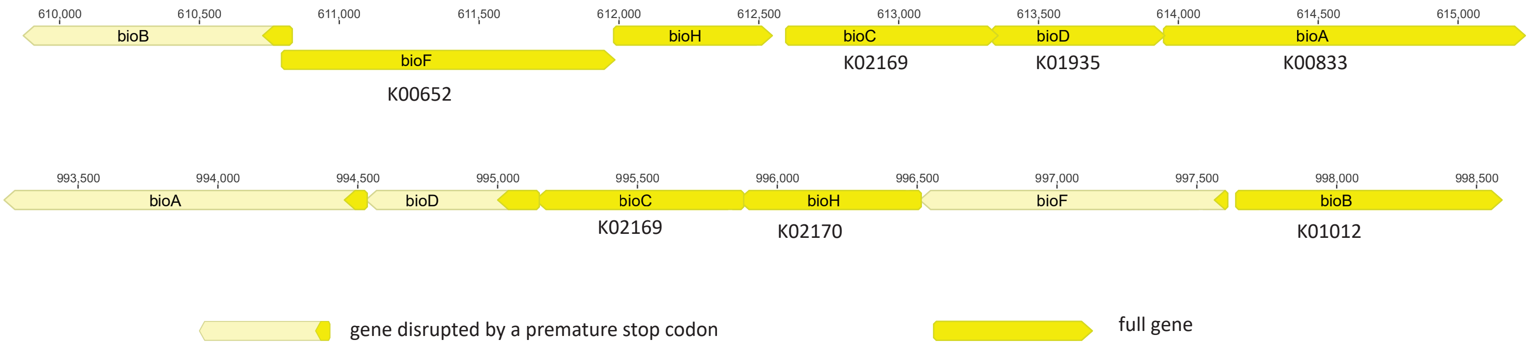

Supplementary figure S3. Two biotin operons in genome of C44-Paracimex with complementary disrupted/functional genes. The numbers above genes indicate positions in the genomes; the assigned K numbers indicate which genes were recognized by BlastKoala as functional.

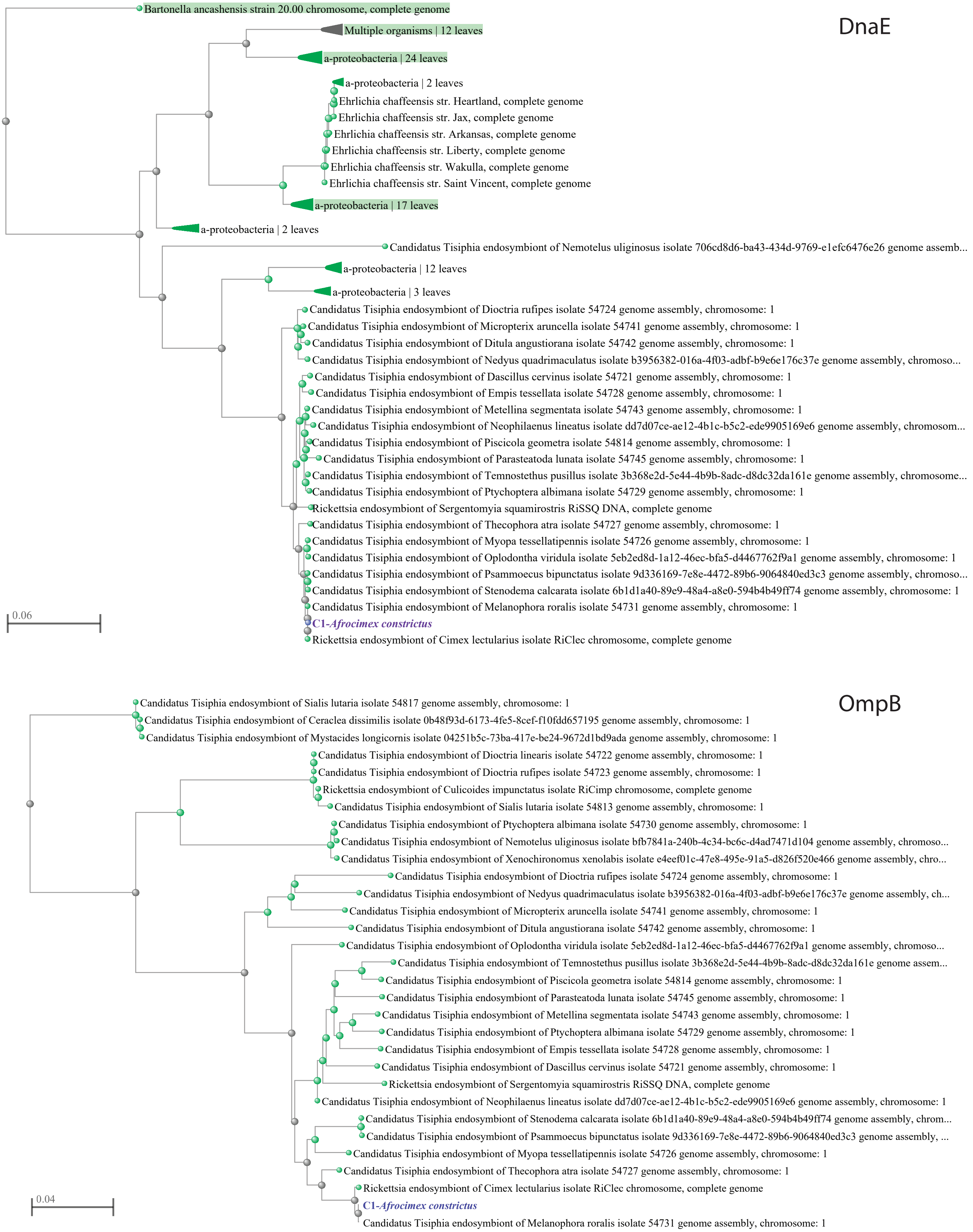

Supplementary figure S4. Phylogenetic trees inferred using NCBI-based fast minimum evolution algorithm, based on two genes (DnaE and OmpB) from fragments of the *Tisiphia* genome extracted from the C1-*Afrocimex constric-tus* metagenome.
